# Finnish late adolescents’ physical activity during COVID-19 spring 2020 lockdown

**DOI:** 10.1186/s12889-021-12263-w

**Published:** 2021-12-01

**Authors:** Kwok Ng, Pasi Koski, Nelli Lyyra, Sanna Palomaki, Kaisu Mononen, Minna Blomqvist, Tommi Vasankari, Sami Kokko

**Affiliations:** 1 School of Educational Sciences and Psychology, University of Eastern Finland, Joensuu, Finland; 2 Physical Activity for Health Research Cluster, Department of Physical Education and Sport Sciences, University of Limerick, Limerick, Ireland; 3 Department of Teacher Education, University of Turku, Rauma, Finland; 4 Faculty of Sport and Health Sciences, University of Jyvaskyla, Jyvaskyla, Finland; 5 KIHU Research Institute of Olympic Sports, Jyvaskyla, Finland; 6 UKK Institute for Health Promotion Research, Tampere, Finland; 7 Faculty of Medicine and Health Technology, Tampere University, Tampere, Finland

**Keywords:** Organised sport, Physical education, Walking, Cycling, Home-gym

## Abstract

**Background:**

Physical activity (PA) is recognised as one of the leading and effective strategies to prevent non-communicable diseases that boosts the immune system to fight against diseases. Closures of schools, sport clubs and facilities because of COVID-19 reduced the opportunities to participate in PA. We aimed to examine physical activity levels of late adolescents, the contexts to be physical active and its changes during the spring 2020 lockdown.

**Methods:**

A national representative sample of late adolescents in general upper secondary school (*n* = 2408, females = 64%, mean age = 17.2y, SD = 0.63) completed self-report online surveys on PA behaviours between March and June 2020. Multinominal logistic regression analyses were performed to identify correlates with PA, and decision tree analyses to ascertain the perceived changes on PA during lockdown based on sport club aspirations and levels of PA.

**Results:**

Among the late adolescents, the distribution of PA frequency was 23% (0-2 days/week), 35% (3-4 days/week), 30% (5–6 days/week) and 12% (7 days/week), and differences between males and females were not statistically significant. Participation in both indoor and outdoor PA were 50 times more likely to report daily PA (OR = 54.28, CI = 15.16–194.37) than non-participation. A quarter of late adolescents were not part of a sports club, yet their PA levels increased. Although sports club members generally perceived they did less PA during lockdown, over a third of sport club members with competitive aspirations reported daily PA.

**Conclusions:**

Overall, most late adolescents reported their PA levels decreased during lockdown. Findings from this study continue to demonstrate factors associated with PA in the context of the COVID-19 lockdown.

**Supplementary Information:**

The online version contains supplementary material available at 10.1186/s12889-021-12263-w.

## Background

Physical inactivity is detrimental to the short- and long-term health of adolescents. There is evidence that increased physical activity (PA) boosts the immune system to fight infections and viruses [1], as well as prevention of leading non-communicable diseases [2]. Despite these known advantages, adolescents struggle to maintain a physically active lifestyle and PA levels drop as age increases [3]. Late adolescents (15–20 years old), is a phase where academic results are taken more seriously [4] and competitive sports make participation more exclusive [5]. More barriers to being physical active appear during late adolescents, as individuals experience physical, psychological and social changes [6]. Few studies have targeted this age group exacerbated by the lack of evidence in the correlates and determinants for PA for this age group [7]. Therefore, more observational studies are needed to understand the complex nature of late adolescents’ PA behaviours.

Much of the independence was taken away from adolescents when the COVID-19 restrictions closed down schools, sport practices and social events, known as lockdown. During lockdown, much of late adolescents’ time spent was indoors and PA levels reduced [8]. Yet, a proportion of late adolescents had positive experiences during lockdown with increased freedom to carry out studies or PA in their own time [9]. Despites the challenges, maintenance of PA levels was observed by some during the spring lockdown [10], although typical behavioural patterns were different. For example, Vasankari and colleagues [11] noticed a shift in the time of day for when PA shifted to later in the morning and this was explained by the absent of the commute to schools.

On the 13th of March, the Finnish government declared the use of the Emergency Powers Act, whereby public spaces such as libraries, swimming halls and other public sports facilities were shut down until further notice [12]. Public gatherings were limited to maximum of 10 people, hence the majority of sports clubs and associations seized to function. Schools adapted to distanced learning on the 18th of March [13]. There was a gradual dismantling of these restrictions from the 14th of May, including the possibility for late adolescents to return to school and participation in outdoor sport practices [14].

In Finland, late adolescents aged 16y to 19y have the choice to attend general upper secondary education or vocational college for initial vocational education [15]. General upper secondary education normally lasts 3 years, prepares for students in higher education, and studies are organized in 6-week blocks. The structured physical education (PE) lesson is dependent on the curriculum and is typically two blocks during the 3 years. Although PE is not the only source for overall physical activity, it can be successful in promoting PA levels among individuals who do not typically have PA related leisure time activities [16]. During lockdown, the change of environment for PE was challenging for many. Physical educators experienced difficulties in remote teaching PE [17], and for example in Norway, almost a quarter of PE teachers reported their subject had a lower priority during lockdown [9]. Teachers had little time to prepare for online classes, and most have little or no experience in their preparation for teaching PE remotely [18] which may have limited the common positive effect of PE on PA.

Similarly, sport clubs are common settings for PA and were shut down during lockdown. Reduced levels of PA were common during COVID-19 restrictions because there were reduced opportunities to access coaching, equipment, facilities and others to practice with [8]. For adolescents involved in organised sports, emotional struggles were commonly reported, especially as the competitive season came to a sudden halt or the sensation of loneliness was felt [19]. There were further concerns that adolescents’ physical, mental and social health would suffer from a lack of regular participation [20]. Fast solutions were found, when sport coaches and their athletes would connect remotely to continue with training during lockdown [21], although it remains uncertain the effect on overall PA.

Based on the behaviour epidemiological framework for studying physical activity and COVID-19 [22], the aim of the study is to identify factors associated with PA. Given that three main environments for adolescents to be physically active during lockdown; home, remote schooling and remote sport clubs, the purpose of this study was to describe the levels of PA during lockdown, how remote PE, sport club activities and self-organised PA were correlated with PA among Finnish late teenagers who attended general upper secondary school, and how perceived changes in PA were associated with behaviour during lockdown.

## Methods

### Study design

**Fig. 1:**
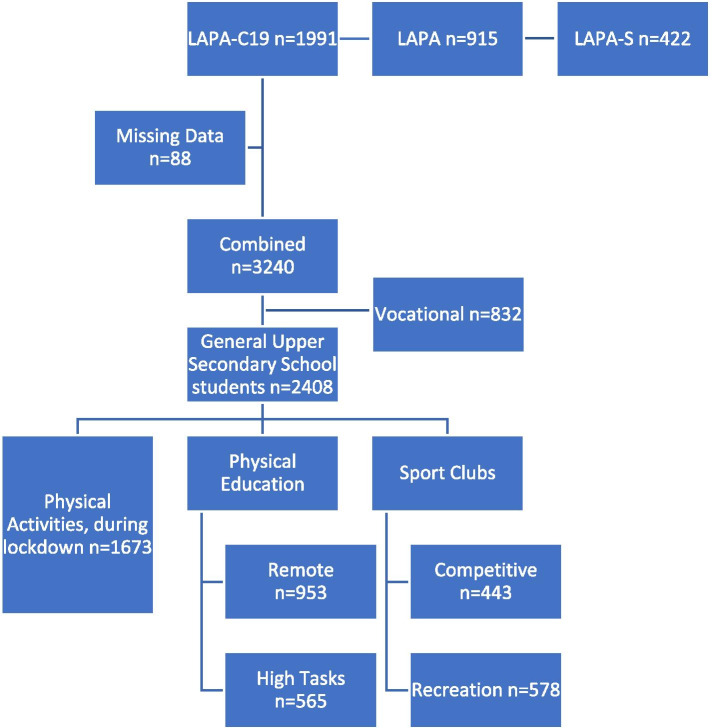
Sample population flow chart for analyses. Late Adolescents Physical Activity (LAPA), COVID-19 (C19), Swedish version (S)

The Finnish Late Adolescents Physical Activity (LAPA) study is a national monitoring physical activity study of late teenagers. It is the extension to the Finnish School-age Physical Activity (F-SPA) study that included 7-, 9-, 11-, 13-, and 15-year olds to late adolescents aged between 16 and 19 years old. The design of the sampling and administration of F-SPA and LAPA were similar. In short, the sampling strategy was based on national representative sample of general upper secondary school and vocational colleges through probability proportion to size, where clusters were set at macro regions of Finland, notably Metropolitan, South, Central and North. A complex sample design included the allocation of survey responses from general upper secondary schools as well as vocational colleges. For the 2019–2020 academic year, there were a total of 105,200 students attended upper general school and 168,000 students attended initial vocational education [15]. The planned data collection phase in spring 2020 commenced on the 9th March. As the emergency powers act came into force on the 17th March 2020, with schools closed on the 18th March, the research team rapidly updated the items in the LAPA study and included items that focused on relationships between PA behaviour and COVID-19 in the LAPA-C19 study. Data from the LAPA-C19 study were collected between 6th April – 5th June 2020. The LAPA study has a split sample design, so that traditional monitoring would continue with the original LAPA study in both Finnish (LAPA) and Swedish (LAPA-S) speaking schools including the collection of week-long accelerometer data. Data from late adolescents in general upper secondary school who completed LAPA-C19, LAPA, and LAPA-S were combined for the purpose of this study (Fig. 1). Contact with late adolescents in vocational colleges were an administrative challenge and for that reason, data from vocational colleagues were removed from the data analyses to avoid biasing the representativeness of the sample. Furthermore, accelerometer data was removed from the data set due to complications to collect representative sample that followed from the COVID-19 restrictions, leaving representative general upper secondary school self-report data in this study.

All surveys were completed anonymously and voluntarily through an online survey. In Finland, late adolescents over the age of 16 have the legal right to consent for themselves to take part in the study. Permission was obtained by all participants in this study. LAPA study was approved by the University of Jyväskylä research ethics committee.

### Survey items

#### Background variables

Age was calculated from respondents input of their day, month and year of birth in relation to the date of survey completion. Age categories were created based on the nearest whole year for 16y, 17y, 18y olds. Disabilities were calculated through the self-reported version of the child functioning module of the Washington group on disability studies [23]. There are 11 items with a four-point scale on core functions for adolescents namely, seeing, hearing, speaking, walking, concentrating, learning, self-care, remembering, change of routines, getting friends, controlling own behaviour. A first past the post system for difficulties at the level of ‘a lot of difficulty’ was used to determine estimates for people with disabilities [24]. Items in relation to social economic status (SES) were determined by two items on parent’s highest level of education and the family finance level. Place of residence was a single item, those who live in a city was coded as urban, and those who live outside of the city were coded as rural.

#### Physical activity variables

A definition of physical activity intensity was included at the beginning of the survey, followed by a single item measure the number of days in the past week where the individual had participated in at least 60 min of moderate to vigorous physical activity (MVPA). This item has been used extensively for national monitoring purposes [25] with good validity against accelerometers [26] and acceptable test-retest reliability [27]. The days were grouped into 0–2 days, 3–4 days, 5–6 days, and 7 days, as 4 categories have meaningful interpretations [28] as well as providing more insight into the ‘every move counts’ connection from the updated WHO PA guidelines [29]. An additional definition of vigorous intensity PA (VPA) followed and a single question on the number days in a usual week the individual does VPA. This variable dichotomised into less than 3 days and more than 3 days as part of the PA guidelines for strengthen exercises [29].

Frequency of 21 physical activities carried out during a week was based on a frequency scale from Never to daily. Items were selected from Finnish expert group and included; muscular fitness training, body care (i.e. stretching/yoga), indoor aerobic, dance, gymnastics, hula hoops, cycling, skating or scootering, jogging, walking or Nordic walking, hiking, orienteering, geocatching, frisbee golf, stair running, walking the dog, skiing or ice skating, nature-based exercises, indoor e-games, outdoor e-games, and other. Respondents who reported ‘other’, wrote in an open-ended response box to describe what was the activity they did during lockdown. These items were cross-checked with the 21 items to identify extra activities that were not on the 21 activities already listed. This resulted in four more activities, ball sports, martial arts, horse riding and golf. Recoding of responses values were never, less than weekly or weekly as 0, and more frequently or daily as 1. The activities were then grouped into no PA, indoor only, outdoor only, and both indoor and outdoor PA.

A separate item was used to measure the extent of change in PA as a result of the emergency measures. The response option was a five-point scale from Much less to Much more with the midpoint [3] as the same. There has not been time to validate the measure, although similarly worded items have been used in a variety of published COVID-19 related papers [30].

#### Physical education

Students were asked if they received PE instruction during lockdown through remote methods. If they reported yes, the respondents were asked their level of agreement (1 completely agree – 4 completely disagree) on whether they completed all the PE tasks during the day. Students who reported ‘completely agree’ were grouped as ‘high task’, all others were grouped as ‘low task’, and students without any remote PE were coded as ‘no remote PE’ as the reference category.

#### Sport clubs

Three groups were created based on the combination of two items, 1. Membership of sport clubs before lockdown, and 2) Level of competitive aspiration. Aspirations for competitive sports for youth and adults were combined into a group called competitive member. Responses of membership but no competitive aspiration were grouped into recreational member and the final and reference group were the responses of no sport club member.

### Statistical analyses

Chi-square test of independence on the proportions for the background variables were carried out to confirm analyses for pooled data from the survey types (LAPA-C19, LAPA, LAPA-S) as well as segmentation analysis (SES and PE) that could be generalised for the rest of the sample. When proportions of MVPA did not differ between the extra responses from LAPA-C19 with the rest of the sample (*p* > .05), the combined data were consisted sufficiently general for the items on PE and SES. Multinominal logistic analyses of the background variables and correlates were performed to identify the associations with different proportions of MVPA categories (3–4 days, 5–6 days and 7 days) to the reference category of 0–2 days.

A decision tree analyses approach through Chi-square Automatic Interaction Detector (CHAID) analysis was used for testing the probability of 0–2 days, 3–4 days, 5–6 days or 7 days of MVPA based on involvement in sport clubs, competitive aspiration and perceptions of change to MVPA. CHAID analysis can determine the probability of each possible node for different frequencies of MVPA.

To estimate the correlates of change in PA, univariate analysis of variance was conducted where change of MVPA was the independent variable and gender, age, disability, place of living, SES, PE, types of activity, frequency of MVPA, VPA and sport club aspirations as dependent variables. IBM SPSS 27.0 with 2-tailed tests and 95% confidence intervals was used for all analyses.

## Results

Most students in Table 1 were female (64%) with the overall mean age of 17.2y (SD = 0.63). Under a quarter (23%) of adolescents reported they were inactive (0–2 days), over a third reported 3–4 days (35%), another third reported 5–6 days (30%), and one in 10 reported they were physically active daily (12%). Almost a half of the students did not have remote PE (43%), a quarter had remote PE but did not always do the tasks (23%), and a third always did the PE tasks (34%). Over a half (54%) of the adolescents reported they did over 3 days a week of vigorous exercise or were not members of organised sports (56%), with over a quarter (26%) doing recreational organised sports and less than one in five (18%) had competitive aspirations in organised sports. Over a third reported they did less (39%) or did more (39%) than normal PA during the lockdown, the remainder (23%) reported the same.

**Table 1:** Sample characteristics by survey type with Chi-square tests of independence

		Total	LAPA-C19	LAPA	LAPA-S	*p*
Gender	Total	2408	1678	363	367	0.065
	Female (%)	63.9	65.4	60.1	60.8	
	Male (%)	36.1	34.6	39.9	39.2	
Age Categories	Total	2408	1678	363	367	0.001
	16y (%)	9.3	9.5	11.6	6.3	
	17y (%)	54.1	55.3	53.7	49.0	
	18y (%)	36.5	35.2	34.7	44.7	
Disabilities	Total	2408	1678	363	367	0.129
	Without Disabilities (%)	84.9	83.7	86.5	88.6	
	With Disabilities (%)	15.2	16.3	13.5	11.4	
	∙ Sensory (%)	2.7	2.8	3.3	1.4	0.212
	∙ Physical (%)	0.4	0.4	0.3	0.5	0.852
	∙ Cognitive (%)	7.3	7.7	6.6	5.7	0.349
	∙ Behavioural (%)	9.1	10.1	5.8	7.6	0.021
Urbanicity	Total	2402	1672	363	367	<.001
	Rural (%)	31.9	31.0	21.5	46.0	
	Urban (%)	68.1	69.0	78.5	54.0	
Change in PA	Total	2305	1652	302	351	.356
	Less (%)	38.6	37.7	39.4	41.9	
	Same (%)	22.5	22.6	24.8	19.7	
	More (%)	39.0	39.6	35.8	38.5	
Family Finances*	Total	1672	1672			n/a
	Bad or very bad (%)	5.0	5.0			
	Average (%)	23.8	23.8			
	Good (%)	51.3	51.3			
	Very good (%)	19.9	19.9			
Remote PE*	Total	1676	1676			n/a
	No remote PE (%)	43.1	43.1			
	Low tasks remote PE (%)	23.2	23.2			
	High task remote PE (%)	33.7	33.7			

*LAPA* Late Adolescents Physical Activity, *C19* COVID-19 version, *S* Swedish version

^*^Only in LAPA-C19 survey

### Physical activity behaviour during lockdown

Half of the late adolescents (52%) reported taking part in both in- and outdoors physical activities at least a few times a week and a quarter (26%) reported participation in outdoors activities. One in 10 reported only indoor (11%) or no physical activities (12%) during lockdown. Walking, muscle strengthening activities, body conditioning (i.e. yoga), running, taking dog for a walk or cycling were common activities during lockdown. Females reported statistically significantly more walking, body conditioning, walking the dog, dancing, gymnastics, hiking and doing hula hoops than males. More males reported to do cycling, frisbee golf, skating or scootering, or mobile physical activity games outsides than females (Supplementary Table 1).

Higher SES was associated with increasing MVPA, but not age and gender (Table 2). Participants in both indoor and outdoor activities during lockdown were over 50 times more likely to report daily MVPA than students who reported no activities and 0–2 days of MVPA (OR = 54.28, CI = 15.16–194.37). The association strength of MVPA decreased with outdoor or indoor activities respectively. There were positive associations between students with competitive aspirations or doing at least 3 days of VPA with increasing days of MVPA compared with 0–2 days and non-participation in organised sport or less than 3 days of VPA. Students who reported high PE tasks were more likely to have taken part in 3–4 days (OR = 1.61, CI = 1.12–2.32) or 5–6 days (OR = 1.84, CI = 1.22–2.78) of MVPA but not daily MVPA compared with 0–2 days and to those not having remote PE.

**Table 2:** Descriptive Multinominal Regression of MVPA from 0 to 2 days MVPA (as reference)

REF 0–2 days	3–4 days				5–6 days				7 days			
	OR	LCI	UCI	*p*	OR	LCI	UCI	*p*	OR	LCI	UCI	*p*
Gender												
Female	1.00				1.00				1.00			
Male	0.857	0.632	1.163	0.322	0.970	0.678	1.387	0.866	0.912	0.579	1.438	0.692
Age (increasing)	0.973	0.765	1.236	0.820	1.184	0.895	1.566	0.236	0.914	0.641	1.304	0.622
SES (increasing)	1.287	1.068	1.553	0.008	1.273	1.021	1.587	0.032	1.586	1.193	2.106	0.001
Disabilities												
Without	1.00				1.00				1.00			
With	0.722	0.499	1.044	0.083	0.627	0.395	0.996	0.048	0.685	0.371	1.264	0.226
Residence												
Rural	1.00				1.00				1.00			
Urban	1.225	0.901	1.667	0.196	1.471	1.022	2.118	0.038	0.923	0.589	1.445	0.725
Remote PE												
Without	1.00				1.00				1.00			
Low Task	0.787	0.554	1.116	0.179	0.572	0.371	0.882	0.012	0.352	0.193	0.643	0.001
High Task	1.608	1.117	2.314	0.011	1.837	1.219	2.769	0.004	1.277	0.773	2.107	0.339
VPA												
< 3/week	1.00				1.00				1.00			
= > 3/week	2.292	1.620	3.243	<.001	13.863	9.378	20.493	<.001	36.373	18.824	70.283	<.001
Organised Sport												
None	1.00				1.00				1.00			
Recreation	1.313	0.940	1.835	0.111	1.179	0.793	1.751	0.416	1.250	0.737	2.118	0.407
Competitive	3.210	1.698	6.070	<.001	7.115	3.686	13.733	<.001	14.451	7.057	29.594	0.000
Activities												
None	1.00				1.00				1.00			
Indoor	4.502	2.624	7.726	<.001	4.891	2.196	10.890	<.001	6.181	1.481	25.793	0.012
Outdoor	6.555	4.171	10.301	<.001	13.957	6.820	28.562	<.001	27.323	7.316	102.041	<.001
In- & Outdoor	10.356	6.549	16.376	<.001	27.652	13.766	55.544	<.001	54.277	15.157	194.373	<.001

*REF* Reference category of 0–2 days MVPA, *OR* Odds ratio, *LCI* Lower 95% confidence interval, *UCI* Upper 95%, confidence interval, *SES* social economic status, *PE* physical education, *VPA* vigorous intensity physical activity

### Sport Club participation

**Fig. 2:**
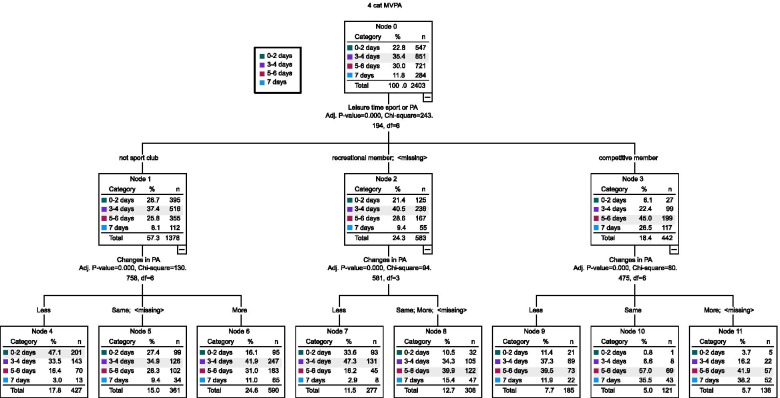
Flowchart of organised sport, changes in PA during spring 2020 lockdown and MVPA levels

A quarter of late adolescents (25%) were not members of sport clubs, yet perceived their PA increased during lockdown. Days of MVPA were strongly associated with participation in sport clubs as well as perceived changes in PA during lockdown. Based on the CHAID analysis (Fig. 2), 47% of students who perceived to do less PA during lockdown and were not involved in sport clubs (Node 4) reported 0–2 days of MVPA. Similarly, 47% of late adolescents who perceived their PA was less during lockdown and were recreational club members (Node 7), 35% perceived the same PA during lockdown as normal and were not in organised sports (Node 5), and 42% perceived their PA levels increased during lockdown and were not in sport clubs (Node 6) reported 3–4 days of MVPA. Students with competitive aspirations, irrespective of perceptions of doing less (Node 9; 40%), the same (Node 10; 57%) or more PA (Node 11; 42%) were most likely to reported 5–6 days of MVPA. Similarly, recreation aspirations and perceptions of doing the same or more PA during lockdown (Node 8; 40%) reported 5–6 days of MVPA and had similar distributions in frequency of MVPA.

### Perceived change in PA during lockdown

**Fig. 3:**
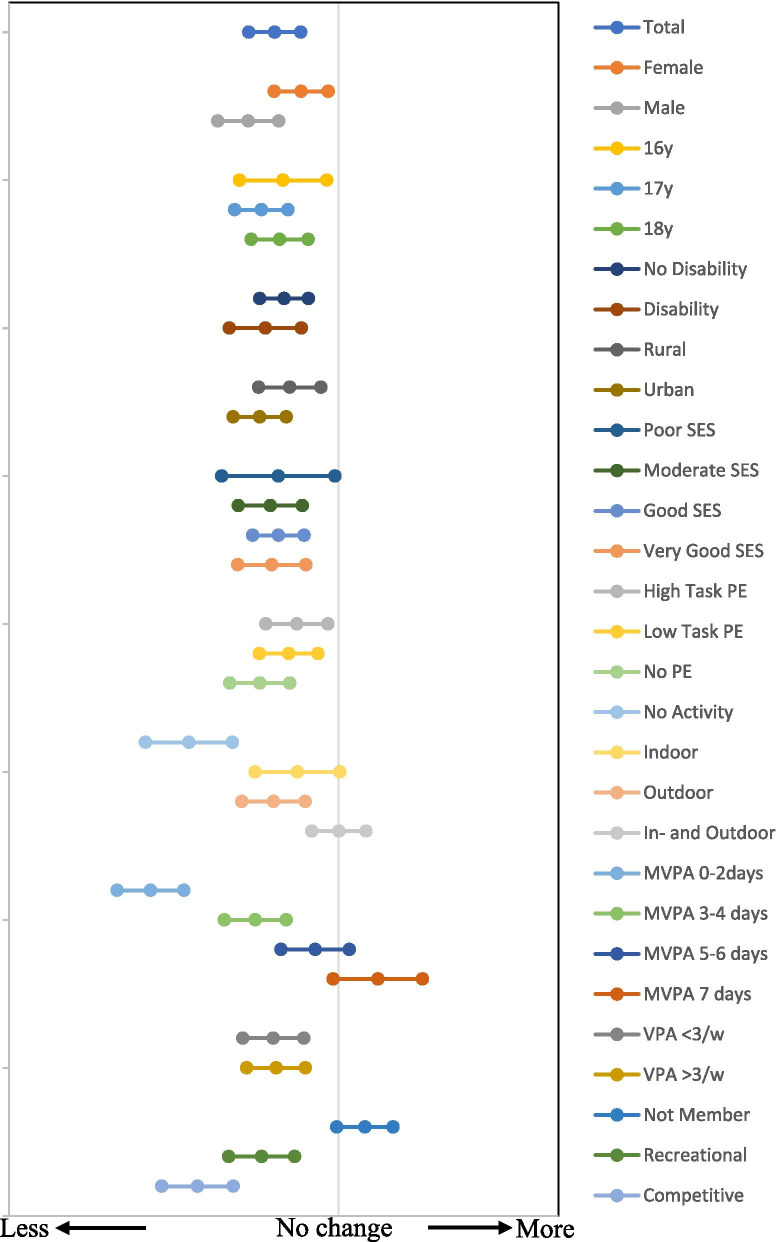
Changes (adjusted mean and 95% CI) in physical activity among Finnish late adolescents during lockdown

From a scale of 1 (less) to 5 (more), late adolescents generally reported to have participated in less PA during the spring 2020 lockdown (mean = 2.71, CI = 2.59–2.83). Males reported their PA levels had reduced more compared to females (2.59 vs 2.83, *p* < .001). In addition, late adolescents who lived in urban places reported their PA levels reduced compared to peers in who live rural places (2.64 vs 2.78, *p* = 0.02). Late adolescent without remote PE were, on average, reporting their PA levels reduced than students who did the tasks from remote PE (2.64 v 2.81, *p* = 0.03). Without participation in any form of PA (2.32), there were perceptions of doing less PA than doing indoor activities (2.81, *p* < .001), outdoor activities (2.70, *p* = 0.001), or both indoor and outdoor activities (3.00, *p* < .001). Late-teens who reported 0–2 days of MVPA felt their PA levels decreased the most (mean = 2.14, CI = 1.99–2.30), followed by 3–4 days (mean = 2.62, CI = 2.48–2.76). Late adolescents who reported daily MVPA felt their PA levels increased during the spring 2020 lockdown (mean = 3.18, CI = 2.98–3.38). The opposite pattern was observed among late adolescents who were not in organised sports, with perceptions of increased PA levels (mean = 3.12, CI = 2.99–3.25), whereas recreational sport club members perceived PA levels reduced (mean = 2.65, CI = 2.50–2.80) as well as members with competitive aspirations (mean = 2.36, CI = 2.20–2.52). In Fig. 3, the forest plot represents the mean values with 95% confidence intervals for changes in PA during lockdown for demographic and physical activity behavioural characteristics. The vertical line represents no change. Mean values to the left represent less PA and values to the right of the line represent more PA during lockdown.

## Discussion

This study examined the associations of PA with the at home school, remote sport club activities, and self-organised PA contexts available to adolescents during the spring 2020 lockdown in Finland. We described the correlates between PA and individual characteristics, PE, sport clubs, and leisure time activities during lockdown, then examined the associations with perceived changes in PA during lockdown. In our sample, increased SES, vigorous PA at least three times a week, competitive aspirations, and indoor or outdoor activities during lockdown were associated with increased levels of PA. Furthermore, a third of late adolescents reported their PA levels were lower than before lockdown and the findings are consistent with previous reports that a large proportion of adolescents reported a reduction in PA [30]. Surprisingly, there was a similar proportion who reported levels of PA during lockdown had increased. These rates were higher than the reported prevalance in earlier studies [8].

In the current study, only 12% of the late adolescents took part in daily MVPA, which is similar to the rates in 2017 for the same age cohorts [31]. Secular trends of daily MVPA may then be unaffected by COVID-19. Despite closures of sport facilities, increased SES was associated with increasing MVPA. Based on the capability, opportunity, motivation and behaviour (COM-B) model and PA, Hankonen and colleagues [32] reported higher SES was associated with self-monitoring, action planning, and greater material resources explaining the possible reasons for being physically active among late adolescents with high SES. Complementary to these results, there were also very strong associations with increases in MVPA with regular VPA, organised sport with competitive aspirations, and leisure time PA. These correlates have been reported in previous studies [21], although the extent of the effect were larger in our study based on data collected during the spring 2020 lockdown. These findings would suggest there is an increasing gap between late adolescents who are physically active and those who were not, as reported globally [30].

During the spring 2020 lockdown, schools in Finland were closed [13]. We found positive associations between high PE task and participating between 3–4 and 5–6 days a week, but not daily MVPA. For some late adolescents, PE may support physically active lifestyles or it may encourage students to take up PA [16]. This could explain the null association with daily MVPA. PE contributes to overall PA, but on its own is not enough to support daily MVPA [33]. From PE lessons, there might be an extra school task that does not contribute to overall PA than a simple exercise [34]. This latter reason could be one reason for the negative association for low PE task and 5–6 days of MVPA and may have been exacerbated through remote teaching [35]. PE teachers encountered technological barriers in the remote teaching space [36], as well as faced a new paradigm to include the family and community into the PE lesson [37].

As a result of closure of schools, sport clubs, and fitness centres, levels of PA reduced and left PA opportunities to be based on individual activities in compliance with physical distancing measures [8, 14]. The lockdown measures were common around the globe and has been reported to reduce the amount of PA by half among adolescents [30]. In our Finnish sample, the most frequently reported outdoor activity was walking. This was an opportunity for late adolescents to meet a friend outside in a safe place. Finns have easy access to nature to go for walks [38] and the outdoor activities were more positively associated with increased levels of PA than indoor activities, yet was not sufficiently practiced by all to prevent reported declines in MVPA. Almost half the respondents reported doing muscle strengthening exercises and a third with body conditioning activities at least a few times a week. These activities may have been done unsupervised or through the support of on-demand coaching or fitness sessions. During the lockdown period, one of the largest fitness chains in Finland offered a schedule of free on demand fitness sessions and this may have been one way for the late adolescents to have done more PA during lockdown. This may have led to increases in PA among the 25% of late adolescents who were not part of a sports club. The uptake to do more PA from non-members of sport clubs could also be related to capacity, knowledge, and more time, as noted in other studies [8]. Further studies are needed to investigate the mechanisms of influencers, remote coaching, its effectiveness as well as the uptake, motives, and capacity to participate in it.

The results from the decision tree analyses confirms the widening of gap between physically active and inactive based on competitive aspiration in sports among late adolescents. In relation to the spring 2020 lockdown, late adolescents with competitive aspirations may have had more possibilities to remote and on-demand training with coaches willing to find ways to keep their athletes in condition [39]. For late adolescents not part of sport clubs, independent exercises such as walking, exercise classes and dance were more common among females than males. The activities were easy to arrange and maybe more attractive for females to do than males who see sports as the main ways to take part in PA [40]. As the opportunities for sport were reduced from the lockdown, it was common for males to turn to online gaming for leisure time activities, and this type of behaviour was typically associated with lower physical activity levels [41].

This study has some limitations for the readers to consider. The instruments reported were self-report and there might be some bias. The instruments used were originally taken from previously validated measures for these participants. Due to the rapid changes from the COVID-19 pandemic, the question about perceived change has not been tested for psychometric properties, as with many other studies that have used the similar measure. The data is cross-sectional; therefore, no causal assumptions can be made. The late adolescents included in this study were from general upper secondary schools, and may not represent all late adolescents as a large proportion attend vocational colleges [42]. It has been challenging to recruit vocational colleges adolescents due to their placement and low levels of in-person contact with their teachers to administer a classroom based survey like this LAPA study. Despite the study limitations, the study has several strengths based on the national representative sample of general upper secondary schools, the unique data collected during lockdown from late adolescents and measures included that are comparable with other surveillance studies.

## Conclusions

Seldom are physical activity levels among late adolescents reported from national representative samples of general upper secondary school students. The differences in PA between Finnish males and females was non-existent, yet commonly reported correlates of MVPA such as SES, vigorous physical activity, sport club, and leisure time activities were associated with increased MVPA. In the context of COVID-19, this paper highlights the changes in PA whereby students who did no leisure time activities, 0–2 days, or were competing in organized sports perceived their levels of PA the least. As reported in Dwyer and colleagues [10], training during lockdown needs to continue to maintain the health benefits from regular PA. Regular accumulation of at least 60 min were most commonly reported to be between 3 and 4 days a week. Furthermore, late adolescents with competitive sport aspirations perceived their levels of PA decreased, although remained the most physically active group.

## Supplementary Information

**Additional file 1: Supplementary Table.** Types of physical activities a few times a week during lockdown by gender. Chi-square test between gender.

## Data Availability

The dataset supporting the conclusions of this article is available upon request with the last author of the manuscript.

## Abbreviations

CI: Confidence intervals
COVID-19: Coronavirus disease 2019
LAPA: Late adolescents’ physical activity
LAPA-C19: Late adolescents’ physical activity COVID-19
LAPA-S: Late adolescents’ physical activity Swedish language
MVPA: Moderate to vigorous (intensity) physical activity
OR: Odds ratios
PA: Physical activity
PE: Physical education
SES: Social economic status
VPA: Vigorous (intensity) physical activity
