# Supplementary material for: Finnish late adolescents’ physical activity during COVID-19 spring 2020 lockdown
Source: BMC Public Health. 2021 Dec 1;21:2197. doi: 10.1186/s12889-021-12263-w (PMC8635322; doi:10.1186/s12889-021-12263-w)
Supplement: Supplementary file 1 — Additional file 1: Supplementary Table. Types of physical activities a few times a week during lockdown by gender. Chi-square test between gender. [file 12889_2021_12263_MOESM1_ESM.docx]

# Supplementary Tables

Supplementary Table. Types of physical activities a few times a week during lockdown by gender. Chi-square test between gender.

|  |  | Female (%, n=1090) | Male (%, n=568) | Total (%, n=1658) | p |
| --- | --- | --- | --- | --- | --- |
|  |  |  |  |  |  |
| Walking |  | 60.4 | 34.8 | 51.7 | <.001 |
| muscle strengthening |  | 44.7 | 46.4 | 45.3 | 0.508 |
| body conditioning |  | 37.0 | 25.7 | 33.1 | <.001 |
| running |  | 30.3 | 28.2 | 29.6 | 0.380 |
| walking dog |  | 31.9 | 19.1 | 27.6 | <.001 |
| cycling |  | 14.5 | 25.8 | 18.4 | <.001 |
| dance |  | 18.5 | 1.6 | 12.8 | <.001 |
| outdoor exercising |  | 11.4 | 12.1 | 11.7 | 0.676 |
| resistance training |  | 12.2 | 9.9 | 11.4 | 0.153 |
| frisbee golf |  | 3.2 | 13.5 | 6.7 | <.001 |
| gymnastics |  | 5.8 | 2.6 | 4.7 | 0.003 |
| hiking |  | 4.7 | 2.4 | 3.9 | 0.022 |
| hula hoops |  | 4.7 | 1.5 | 3.6 | 0.001 |
| steps running |  | 3.6 | 2.9 | 3.4 | 0.441 |
| skate or scootering |  | 1.8 | 5.6 | 3.1 | <.001 |
| sport console games |  | 2.9 | 1.5 | 2.4 | 0.070 |
| esport games outside |  | 0.9 | 2.9 | 1.6 | 0.003 |
| skiing or skating |  | 1.3 | 2.0 | 1.6 | 0.277 |
| Geo-catching |  | 0.3 | 0.7 | 0.4 | 0.194 |
| orienteering |  | 0.4 | 0.4 | 0.4 | 0.976 |
